# Mental distress, COVID19 vaccine distrust and vaccine hesitancy in South Africa: A causal mediation regression analysis

**DOI:** 10.1371/journal.pone.0278218

**Published:** 2023-03-24

**Authors:** Umakrishnan Kollamparambil, Adeola Oyenubi, Chijioke Nwosu

**Affiliations:** 1 School of Economics & Finance, University of the Witwatersrand, Johannesburg, South Africa; 2 The Impact Centre, Human Sciences Research Council, Cape Town and University of Free State, Bloemfontein, South Africa; The University of the West Indies, TRINIDAD AND TOBAGO

## Abstract

**Aim:**

Within the context of increasing mental distress noted since the beginning of the COVID19 pandemic, the study aims at analysing the relationship between mental health, vaccine distrust and vaccine hesitancy in South Africa.

**Subject and methods:**

The study uses nationally representative panel data of 3241 individuals interviewed prior to and during the COVID19 pandemic. The study uses a range of regression techniques including logit, mediation and gradient-boosted causal mediation models to identify the causal relationship while accounting for selection bias.

**Results:**

The results of multivariate logit regression reveal that vaccine distrust is the most important predictor of vaccine hesitancy [Coeff: 3.420, CI 3.122, 3.717]. Mental distress is not found to be a significant driver in a fully specified logit model, pointing to the need to analyse the pathways through which mental distress impacts vaccine hesitancy. The mediation regression undertaken for this purpose finds that the mental distress has a positive and significant association with vaccine distrust [Coeff: 0.027, CI: 0.0029, 0.052]. The increased vaccine distrust in turn results in increased vaccine hesitancy [Coeff: 0.661, CI: 0.611, 0.711]. The results of mediation regression therefore indicate strong and significant mediation effects, whereby mental health effects vaccine hesitancy through the mediating variable of vaccine distrust. These results are robust to the gradient boosted causal mediation model which establishes strong and significant indirect effects [Coeff: 0.015, CI: 0.01, 0.019], whereby mental health effects vaccine hesitancy through the mediating variable of vaccine distrust.

**Conclusion:**

The study concludes that mental health impacts on vaccine intention through the mediating role of vaccine distrust. The findings indicate that individuals at high risk of depression are more concerned regarding the safety of vaccines, which in turn feeds into vaccine hesitancy. Therefore, depressive symptoms impact on vaccine hesitancy through the mediating factor of vaccine distrust.

## 1. Introduction

A systematic review and meta-analysis of the psychological and mental health impact of COVID19 has shown increased prevalence of anxiety and depression among the general population [1]. South Africa is no exception, and it is widely acknowledged that the pandemic has resulted in a surge in depressive symptoms in the country [2]. The source of this increase is attributed to the fears and anxiety of getting infected with and death from COVID-19 [3–5], as well as the distress caused through large scale economic devastation and job loss [6].

While there is now acceptance of the mental health impact of the pandemic, the role that depressive symptoms play in driving vaccine behaviour and attitudes is less understood. Understanding this relationship is important to address the mental wellbeing of the population as well as to improve vaccine acceptance, in order to bring the pandemic under control.

According to the World Health Organisation Vaccine Hesitancy Working Group [7], vaccine hesitancy is a complex behavioral phenomenon ‘influenced by factors such as complacency, convenience and confidence’ (3C’s model of vaccine hesitancy). Complacency is driven by low infection risk perception as well as low perceived severity of infection. Confidence is driven by the self-efficacy of being able to avoid infection through non-pharmaceutical interventions or vaccines. Therefore, the perceived distrust of vaccines can lower this confidence. Lastly, convenience is related to the access and cost of vaccination.

The importance of communication and context is now increasingly acknowledged extending the 3 C’s framework to 5 C’s [8]. The level of information as well as source of information plays a role in driving vaccine intention. Lastly, the contextual influences, which may be external as well as personal/individual experience also play a role in driving the decision regarding COVID19 vaccines. While the external reality is constructed from social interactions, culture, religion etc.; individual factors like physical and mental health status also play a role. This study focuses on the role of the latter in driving vaccine behaviour.

Apart from the direct influence of mental health on vaccine behaviour, it is also likely to have an indirect effect on vaccine intention via the role it plays in the subjective risk assessment of the newly developed vaccines for COVID19. The mental health of an individual therefore is expected to play a big role in this process through both direct and indirect means.

Existing studies have explored the role of personal health status in driving vaccine intention primarily through physical health status. Kollamparambil et al. [9] found in the context of South Africa that those with chronic physical health problems are more willing to accept COVID19 vaccines. While various socio-economic correlates of vaccine behaviour have been identified in other country contexts as well [10–12], these studies however did not incorporate the relationship between vaccine behaviour and mental health conditions. Internationally as well, studies on the relationship between mental health and vaccine intention are scarce. Only a handful of studies like Batty et al. [13], Bendau et al. [14] and Paul et al. [15] have examined the issue within the context of the COVID19 pandemic. Buneviciene [16] on the other hand find a significant association between pre-existing health conditions and COVID-19 related mental distress. The study however does not assess the implication of mental health on vaccine attitudes and intentions.

A challenge in studying the relationship between depressive symptoms and vaccine intention is the simultaneity that exists in the direction of causation. While negative attitudes to the vaccine can lead to enhanced depression, a depressed state of mind can also impact on the vaccine perception. Batty et al. [13] accounts for this by assessing the relationship between pre-pandemic mental health and vaccine hesitancy in the context of the UK. The study found that having a pre-pandemic mental health condition was unrelated to the willingness to take up a vaccine; whereas physical health was found to be strongly associated with vaccine uptake. However, the study does not consider the indirect channels of impact, whereby mental health impacts vaccine intention via vaccine attitude.

Similar to Batty et al. [13], Bendau et al. [14] found in the context of Germany that the broader constructs of unspecific anxiety and depressive symptoms were not significantly associated with vaccine acceptance. On the other hand, the study found that COVID19-related anxiety, and fears of infection and health-related consequences correlated significantly positively with vaccine acceptance. In contrast, social and economic fears showed significant negative associations with vaccination willingness. The authors conclude that these findings highlight the need to differentiate between several types of fears and anxiety to predict their influence on vaccine acceptance.

Paul et al. [15] also did not find evidence of the impact of pre-existing mental and physical conditions on vaccine intent in the UK. The study is also one of the few that have examined associations between general vaccine attitudes and intent to vaccinate against COVID19. The study found confidence in vaccine safety to be the largest determinant and concludes that negative attitudes towards vaccines are a major public health concern in the UK. Therefore, the study concludes that public health messaging should be tailored to address these concerns and specifically to women, ethnic minorities, and people with lower levels of education and incomes.

Similar conclusions are arrived at by Thunstrom et al. [17] in the context of the US. Thunstrom et al. does not include the mental health variable in their analysis but the study finds general mistrust in vaccines and concerns about future side effects in particular to be barriers to achieving population immunity to COVID19 through vaccination.

The existing literature looks at the direct effect of mental health on vaccine intention, but do not assess the indirect impact mental health may have on vaccine intention via the mediating role of vaccine distrust. Further, the limited number of existing studies exploring the role of mental health have been conducted in high-income countries. This study therefore adds value on two fronts; a) it provides a developing country perspective with high level of poverty and inequality and, b) it goes beyond the direct relationship between mental health and vaccine intent, by exploring the indirect channel of vaccine attitude through which mental health can drive vaccine behaviour.

## 2. Methods

This study is primarily based on Wave 5 of the nationally representative National Income Dynamics Study—Coronavirus Rapid Mobile Survey (NIDS-CRAM) survey. The survey investigates the socioeconomic impacts of the national lockdown associated with the State of Disaster declared in South Africa in March 2020, and the social and economic consequences of the global Coronavirus pandemic [18].

NIDS-CRAM is a special follow up with a subsample of adults from households in the National Income Dynamics Study (NIDS) Wave 5 (2017). In addition to NIDS-CRAM, we also use the data for pre-pandemic mental health from NIDS wave 5 survey. We also supplement this, in a limited way, with data from NIDS-CRAM waves 1 and 2 when required by the analysis.

Though the NIDS-CRAM sample is drawn from the NIDS respondents, the former has a smaller sub-sample of adults compared to NIDS. The NIDS-CRAM questionnaire is also much shorter compared to NIDS questionnaire. The focus of the NIDS-CRAM questionnaire is on the Coronavirus pandemic and the national lockdown. The mode of the NIDS-CRAM survey is Computer Assisted Telephone Interviewing (CATI) surveys, repeated over several months in the preferred official South African language of the respondent. Wave 5 of the NIDS-CRAM survey had 5,862 individuals successfully interviewed over the period 6 April-11 May 2021. Wave 1 and 2, which are also used to a limited extend were undertaken during the period 7 May-27 June 2020 and; 13 July- 13 August 2020 respectively.

The study derives data on mental distress from the 2-question version of the Patient Health Questionnaire (PHQ-2, which is the abbreviated version of the widely used PHQ-9 [19]), viz., “*Over the last 2 weeks*, *have you had little interest or pleasure in doing things*?” and “*Over the last 2 weeks*, *have you been feeling down*, *depressed or hopeless*”. The response to both questions could be “*not at all*”, “*several days*”, “*more than half the days*” or “*nearly every day*”. The responses are coded from 0 to 3, resulting in a depressive score ranging from 0 to 6. The increasing values of the PHQ-2 variable indicates a higher risk of depression. The best cut-off for determining depression remains contested. While some studies [20, 21] identify the PHQ-2 threshold of ≥ 2 as providing the ideal balance between sensitivity and specificity, others suggest a cut-off of ≥ 3 as optimal [19]. We use both thresholds in the analysis. We also use the depressive score variable as a continuum of distress to navigate the uncertainty relating to threshold determination [22–25], which have been found to vary across different language groups in South Africa [26].

Pre-pandemic mental health is captured using the ten items on the Centre for Epidemiological Studies Depression (CESD-10) scale from NIDS wave 5. Each question could be responded to as “*not at all*”, “*several days*”, “*more than half the days*” or “*nearly every day*”. The responses are coded from 0 to 3, creating the outcome variable of CESD-10 scale with a range of 0 to 30, with increasing values indicating higher risk of depression.

Vaccine intention is derived from wave 5 of NIDS-CRAM question, “*To what extent do you agree or disagree with the statement*: *If a vaccine for COVID-19 were available*, *I would get it*?”, allowing for response on a four-point scale of “strongly agree, somewhat agree, somewhat disagree and strongly disagree”. For the purposes of binary variable creation for vaccine hesitancy, responses of ‘somewhat disagree and strongly disagree” are considered to vaccine hesitant; while responses of “strongly agree, somewhat agree” are considered as vaccine willing.

Vaccine attitude is derived from two questions, “Do you believe that the vaccine is unsafe or could harm you?” (with response options of: yes/no) and “How convinced are you of this?” (with response options of: a little/somewhat/very convince). Using these questions we create an ordinal variable ranging 0–3, where 0 indicates belief in the safety of vaccine and 3 being very convinced of lack of safety.

In order to imbibe the severity of risk we additionally use two proxy variables viz., older age groups as well as those with pre-existing chronic illnesses [9]. Therefore, the first proxy for severity (mortality threat) incorporated in the model is age (measured in years) and the second proxy is obtained from wave 1 of NIDS-CRAM survey which included the question “Do you have any of these chronic conditions (you don’t have to tell us which one): HIV, TB, lung condition, heart condition or diabetes?”. This question was not repeated in subsequent NIDS-CRAM waves.

In order to control for the awareness level of individuals, a binary variable is constructed which takes the value 1 if the respondent is aware of the three most important symptoms of infection (cough, breathlessness and fever). The assumption made is that a person who is fully aware of the COVID19 symptoms would also be well-informed of the vaccine [9]. The source of information is also included with binary variables capturing information sources as social media, news, government and health worker. Both the awareness of COVID19 symptoms and source of information were collected only in wave 1 during May/June 2020 and as such is limited by the time lapse between the survey waves. More generally, education is also considered as a proxy for awareness.

Lastly, the convenience factor is incorporated into the model using household income based on the argument that income enables easier access and transport to vaccine centres. The household income variable has a high proportion of missing information in the survey data. As such, rand value responses to household income variable have been supplemented with the median value of the income bracket responses. This approach can however distort the income distribution and therefore following Wittenberg [27] we reweight those who provide rand amounts using the inverse of the probability that an individual will report a rand amount in that bracket. Although this approach does not account for observations that have both the rand value and income bracket information missing, we are able to retain 5,468 out of 5,862 observations.

Because individual responses emerge from the context of different social, cultural, political and personal factors in vaccine decision [28], additional socio-economic variables such as sex(male), race (black African), partnered, location (urban) and religiosity are incorporated to get a clearer picture of the range of possible predictors about vaccine intention.

Further limitation of the data used is that it is largely self-reported and therefore hard to assert that there was no strategic bias in response. Therefore, the study acknowledges the limitation that it may be susceptible to hypothetical and strategic bias [29] especially for key questions like vaccine intention and vaccine distrust.

The NIDS-CRAM survey received ethical clearance from the University of Cape Town Commerce Ethics Committee (REC 2020/04/017) and reciprocal ethics from Stellenbosch University. All participants in the survey provided informed consent. The data was collected through a telephonic survey undertaken during COVID19 lockdown context, hence consent obtained was verbal. The ethics committees approved this consent procedure.

In order to identify the drivers of vaccine hesitancy and more specifically the role of mental health, we first undertake a baseline estimation of a logit model. The baseline multivariate logit regression function to be estimated will be:

$${Vac\_H}_{i}=\beta_{0}+\beta_{1}({Vac\_D}_{i})+\beta_{2}({Pre\_Dep}_{i})+\beta_{3}({Covid\_Dep}_{i})+\gamma X_{i}+\varepsilon_{i}$$ (1)

where:

Vac_Hi denotes vaccine hesitancy taking the value 1 for person i who is unwilling to accept vaccination, and 0 for others

*Vac_D_i_* denotes vaccine distrust, taking value 0 to 3, with 3 denoting lowest level of trust,

*Pre_Dep_i_* is the pre-pandemic level of CESD-10 depressive symptoms score taking value 0 to 30, with 30 denoting highest risk of depression,

*Covid_Dep_i_* is the PHQ2 depressive symptoms observed currently during the pandemic ranging 0–6

Xi denotes a vector of explanatory variables that include confidence, complacency, communication, convenience and context that characterize individual i and,

*ε*i encapsulates the error term.

**Fig 1:**
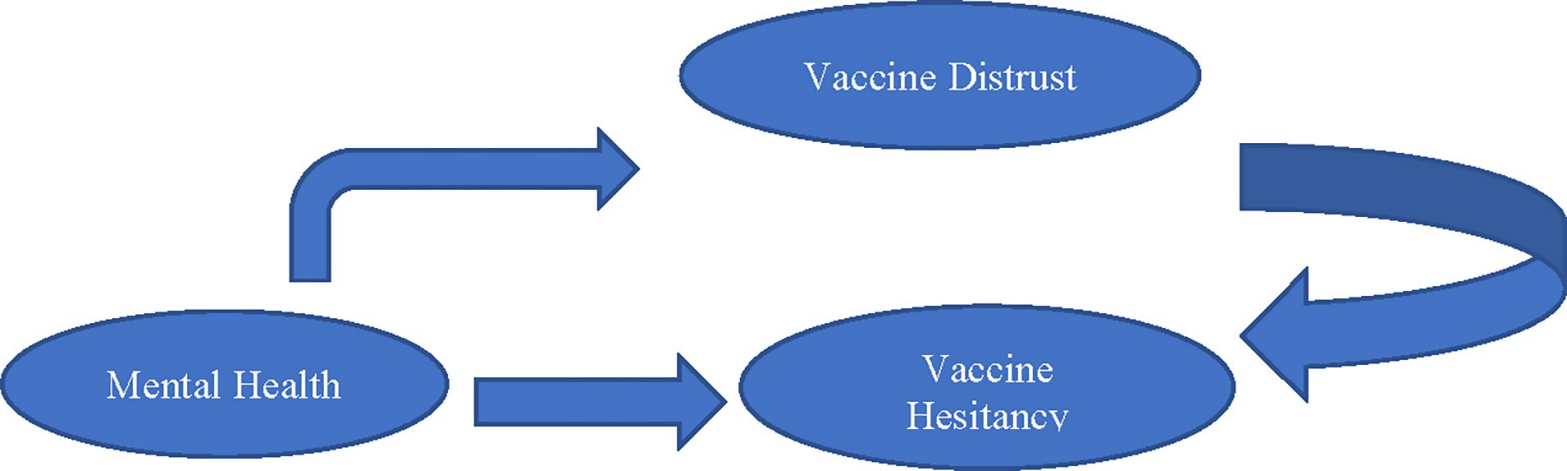
Relationship between mental health, vaccine distrust and vaccine hesitancy.

Following the logit regression, we undertake a mediation regression analysis to identify the pathways through which mental health affects vaccine hesitancy. The direct relationship between mental health and vaccine hesitancy, as well as the indirect relation via the hypothesised mediating role of vaccine distrust is presented in Fig 1.

The mediation analysis is not immune to selection problems, we therefore further consider causal mediation analysis under the conditional independence assumption [30]. Causal mediation analysis estimate mediation effect in the potential outcome framework [31], this approach is similar to the popular causal estimation under ignorability assumption using propensity scores. However, in this case we are interested not only in the total (treatment) effect but the decomposition of this effects that yields the natural direct and indirect effects [32]. The logic here is that the causal effect of mental health on vaccine hesitancy has two causal pathways, there is a direct pathway where mental health directly affects vaccine hesitancy and there is an indirect pathway where the effect of mental health on vaccine hesitancy operates through vaccine distrust (which is the mediating variable).

To operationalize this approach, causal mediation analysis imposes certain assumptions (1) positivity assumption i.e. all individuals have some positive probability of receiving each level of exposure and each level of treatment (2) sequential ignorability assumption which comes in two parts (i) treatment assignment is ignorable given observed covariates (ii) mediator is ignorable given the observed treatment and pre-treatment confounders [31]. Under these assumptions it is possible to identify potential outcomes that allows for the computation of the natural direct and indirect effects. For more details on this methodology interested readers should see Nguyen et al [32, 33], Coffman et al [34], Tingley et al [35] and Imai et al [31].

To make the ignorability assumptions more plausible there is need to balance the distribution of controls across treatment and mediator status. This is achieved through weighting by weights derived from propensity scores. To estimate the propensity scores, we follow the causal machine learning literature [36–38]. Specifically, we use the Gradient boosted model (GBM) Friedman, [39]. GBM is a machine learning approach that depends on decision trees. Being a non-parametric prediction model, it can be used to fit a nonlinear surface and predict group assignments. Its main advantage over regular propensity score models (like logit) is that it allows for flexible, non-linear relationships in estimating propensity scores. As a consequence, it captures non-linear effects and interactions terms without prior knowledge of the model thereby mitigating model misspecification problems. This is important because Propensity score model misspecification has been shown lead to bias in the resulting effect estimate [40, 41]. By automatically considering higher order terms this approach makes the ignorability assumption more plausible. GBM has been shown to be less bias than parametric models[42, 43].

## 3. Data description

**Fig 2:**
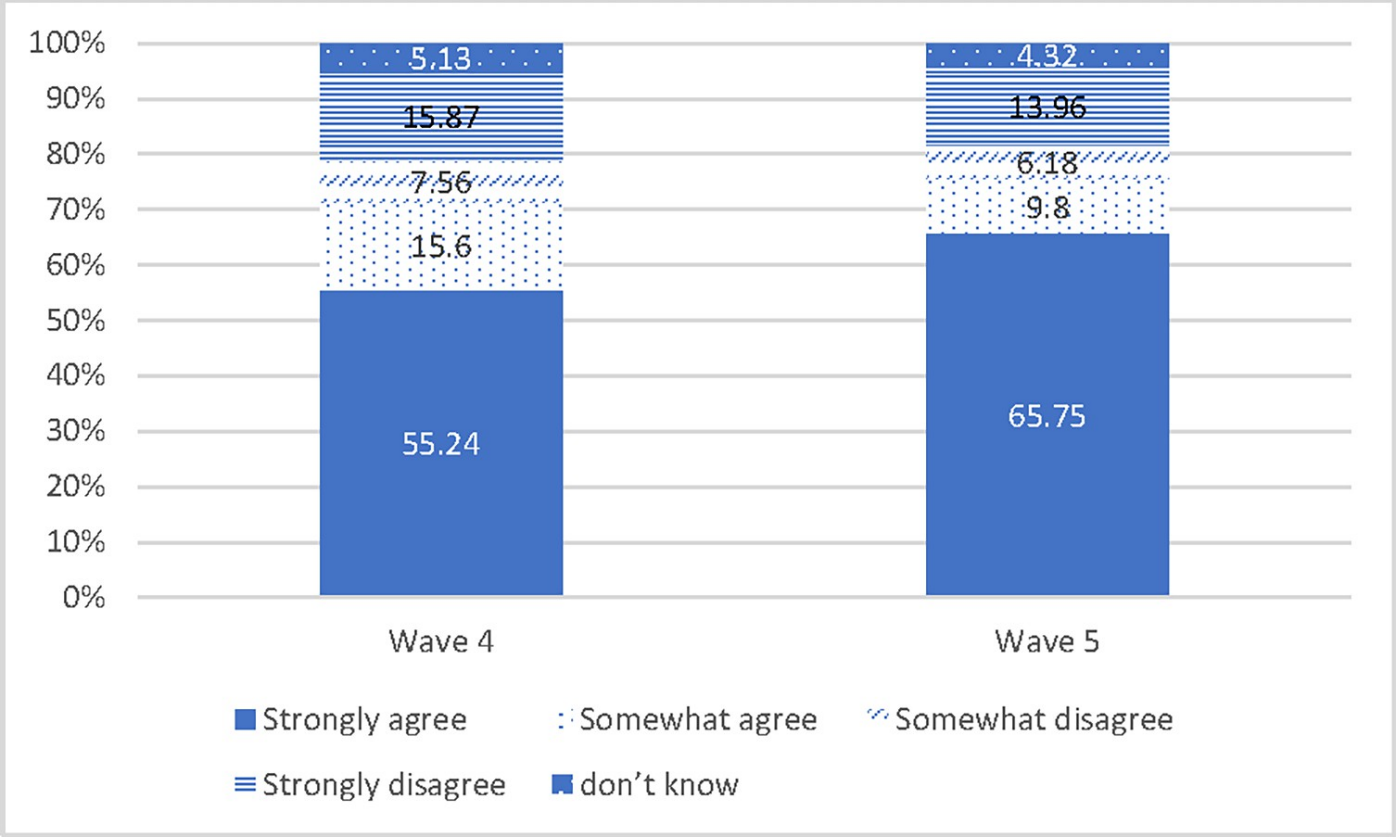
Vaccine intention (%). Source: NIDS-CRAM waves 2 and 5, weighted.

At the time of the survey, only 2% of the population had been vaccinated in South Africa. Vaccine hesitancy is calculated as the proportion of respondents who *strongly disagreed*, *somewhat disagreed* or *didn’t know* about accepting vaccine. Our results show that vaccine hesitancy has declined from 29.2% in wave 4 to 24.5% in wave 5 (Fig 2). Also, there is a substantial shift from those who “somewhat agreed” to “strongly agreed” to the vaccine. This is a good sign; however, hesitancy still remains too high to achieve community immunity especially considering that children are not likely to be included in the vaccination target group.

**Fig 3:**
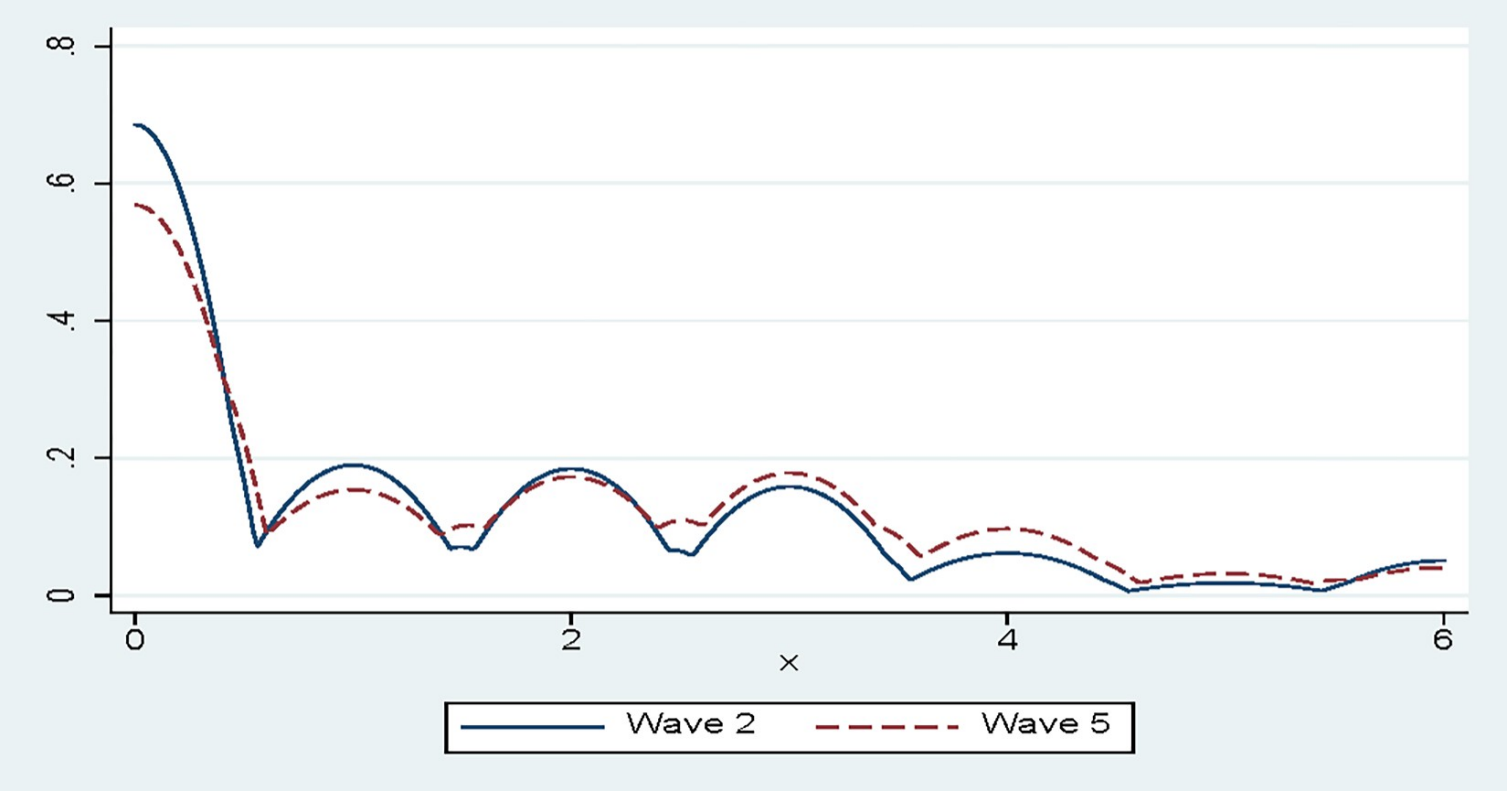
Kernel density plot: PHQ-2 waves 2 and 5. Source: NIDS-CRAM waves 2 and 5, weighted.

The kernel density plots of depressive scores in waves 2 and 5 indicate elevated levels of depressive symptoms with the progression of the pandemic (Fig 3). The average PHQ2 scores increased from 1.294 in July-August 2020 (Wave 2) to 1.487 in April-May 2021 (Wave 5). Using the binary variable of those at risk of depression (with PHQ-2 cut-off of 3), individuals at risk increased from 24.07% to 27.57% between Wave 2 and 5. Using the cut-off of 2, those at risk increased from 38.73% to 42.14%. The increased mental distress observed is despite the fact that the country was in far more relaxed lockdown level in Wave 5 (lockdown level 1) compared to Wave 2 (lockdown level 3). According to Oyenubi and Kollamparambil [44], the increased depressive symptoms, despite lockdown level being relaxed, indicates that the economic recovery would be critical in reviving the mental health of the population.

A brief summary of the various characteristics of our weighted sample of 3241 individuals is provided in Table 1. The mean values point to the sample being nationally representative in terms of the most important demographics like race, sex, gender composition.

**Table 1:** Descriptive statistics.

Variable	Obs	Mean	Std. Dev.	Min	Max
Vaccine distrust	3,241	0.173	0.378	0	1
Pre-Covid mental distress	3,241	6.312	4.277	0	26
Age years	3,241	41.074	14.848	18	98
Above 60 years	3,241	0.127	0.333	0	1
Chronic illness	3,241	0.197	0.398	0	1
Household income pc (log)	3,241	6.833	1.473	0	11.08216
School years	3,241	11.483	3.697	0	22
African	3,241	0.794	0.405	0	1
Male	3,241	0.461	0.499	0	1
Married	3,241	0.469	0.499	0	1
Employed	3,241	0.530	0.499	0	1
Religious	3,241	0.920	0.272	0	1
Hunger	3,241	0.160	0.367	0	1
Social media info	3,241	0.137	0.344	0	1
Government info	3,241	0.140	0.347	0	1
Health worker info	3,241	0.101	0.302	0	1
News info	3,241	0.799	0.401	0	1
Well informed of COVID symptoms	3,241	0.106	0.308	0	1

Source: NIDS-CRAM wave 5, weighted.

## 4. Multivariate regression analysis

The logit regression results presented in model 1 (Table 2) indicate that vaccine distrust is the strongest predictor of vaccine hesitancy. The covid-mental distress variable, proxied through the depressive scores is not statistically significant in model 1. This does not change even when the pre-pandemic depressive scores are dropped from the specification. However, once the vaccine distrust variable is dropped, covid mental distress variable is observed to be significant in model. Pre-pandemic mental distress is also significant in model 3. This points to a significant association between mental distress and vaccine distrust resulting in multicollinearity in models 1 and 2.

**Table 2:** Logit regression results.

	(1)	(2)	(3)
VARIABLES	Vaccine Hesitancy	Vaccine Hesitancy	Vaccine Hesitancy
Vaccine Distrust	3.420***	3.423***	
	(0.126)	(0.126)	
Covid_mental distress	0.00371	0.00409	0.0494*
	(0.0328)	(0.0328)	(0.0259)
Pre-pandemic mental distress	0.0199		0.0238**
	(0.0132)		(0.0105)
Age yrs	-0.00798	-0.00699	-0.0258***
	(0.00631)	(0.00627)	(0.00507)
Above 60 years	0.115	0.0913	0.423*
	(0.269)	(0.268)	(0.221)
Chronic illness	-0.160	-0.158	-0.164
	(0.142)	(0.142)	(0.115)
Log Household income pc.	0.0820*	0.0802*	0.0750*
	(0.0485)	(0.0485)	(0.0403)
School years	-0.0400**	-0.0410**	-0.0281**
	(0.0170)	(0.0169)	(0.0138)
African	-0.771***	-0.737***	-0.787***
	(0.160)	(0.158)	(0.128)
Male	-0.236*	-0.254**	-0.147
	(0.122)	(0.122)	(0.0972)
Married	0.0666	0.0557	0.0586
	(0.119)	(0.118)	(0.0957)
Employed	0.152	0.152	0.0925
	(0.125)	(0.125)	(0.0999)
Religious	-0.00148	-0.0323	-0.118
	(0.222)	(0.221)	(0.174)
Hunger	-0.0444	-0.0392	0.00345
	(0.151)	(0.151)	(0.121)
Social media info	-0.0369	-0.0416	0.219
	(0.182)	(0.182)	(0.141)
Government info	0.0678	0.0574	-0.0477
	(0.170)	(0.170)	(0.137)
Health worker info	0.293*	0.288*	0.138
	(0.168)	(0.168)	(0.137)
News info	-0.0727	-0.0760	-0.0534
	(0.144)	(0.144)	(0.115)
Well-informed	-0.0472	-0.0519	-0.0464
	(0.189)	(0.189)	(0.151)
Constant	-1.440***	-1.309**	0.0444
	(0.528)	(0.521)	(0.425)
Wald chi2(21)	240.23***	264.61***	66.01***
Pseudo R2	0.28	0.28	0.04
Observations	3,241	3,241	3,241

Robust standard errors in parentheses

*** p<0.01

** p<0.05

* p<0.1

Other results emerging are that age and education reduce vaccine hesitancy. Black African population group and those who have experienced hunger have on average lower hesitancy.

Our findings are in line with other studies like Paul et al. [15], Thunstrom et al. [17], Sherman et al. [45], and Williams et al. [46] that suggest that the largest behavioural and attitudinal barrier to receiving a COVID-19 vaccine is the general mistrust against the COVID19 vaccine. While our results are able to pick up a mild association between pre-COVID mental distress and vaccine hesitancy, there is no evidence of current mental distress having a significant association with vaccine hesitancy in a fully specified model. The absence of a strong association between mental distress and vaccine hesitancy is in keeping with evidence from UK and Germany [13–15].

The close correlation between current depressive symptoms and vaccine attitude is clear from the model where the former is significant when the latter is excluded from the model. Therefore, current mental distress may not have a direct effect on vaccine intention but impacts it indirectly via vaccine distrust.

The mediation regression results in Table 3 indicate that the depressive symptoms variable has a positive and significant association with vaccine distrust [Coeff: 0.027, CI: 0.0029, 0.052]. The increased vaccine distrust in turn results in increased vaccine hesitancy [Coeff: 0.661, CI: 0.611, 0.711]. The results of mediation indicate strong and significant mediation effects, whereby mental health effects vaccine hesitancy through the mediating variable of vaccine distrust.

**Table 3:** Mediation regression results.

VARIABLES	Vaccine Hesitancy	Vaccine Distrust
Covid_mental distress	-0.00102	0.0273394*
	(0.0131)	(0.014855)
Vaccine Distrust	0.661***	
	(0.0194)	
Pre-Covid mental distress	0.00215	0.0027097*
	(0.00143)	(0.0016066)
Age yrs	-0.000809	-0.0051613***
	(0.000661)	(0.0007239)
Above 60 years	0.00937	0.0852571***
	(0.0268)	(0.0279059)
Chronic illness	-0.0159	-0.0129404
	(0.0137)	(0.0149583)
Log Houehold income pc.	0.008	0.0045337
	(0.00508)	(0.0060459)
Schooling years	-0.00407**	-0.0009318
	(0.00172)	(0.001867)
African	-0.0935***	-0.0856581***
	(0.0223)	(0.023172)
Male	-0.0240*	0.0000498
	(0.0126)	(0.0143722)
Married	0.0075	0.004442
	(0.0123)	(0.0138489)
Employed	0.0149	0.0003095
	(0.0129)	(0.0147933)
Religious	-0.000614	-0.0267997
	(0.0208)	(0.0282905)
Hunger	-0.00426	0.012377
	(0.0143)	(0.0172454)
Social media info	-0.004	0.0683386***
	(0.0211)	(0.0261003)
Government info	0.00754	-0.0234579
	(0.0179)	(0.0198471)
Health worker info	0.0317	-0.0135239
	(0.0193)	(0.0198064)
News info	-0.00738	-0.0017907
	(0.0159)	(0.017322)
Well informed	-0.00412	-0.0036128
	(0.0196)	(0.0229747)
Constant	0.198***	0.4167355***
	(0.0594)	(0.0694564)
Observations	3,241	3,241
R-squared	0.379	0.0355
F test	70.12***	6.4***

Robust standard errors in parentheses

*** p<0.01

** p<0.05

* p<0.1

The results in Table 3 confirm our initial premise on the mediating role played by vaccine distrust in driving the effect of mental health on vaccine hesitancy. The estimation in Table 3 however cannot claim causal mediation. To address this issue, we next undertake matching through machine learning in the estimation strategy.

The results of causal mediation effect using the gradient boosted model indicates strong and significant indirect effects [Coeff: 0.015, CI: 0.01, 0.019] (Table 4), whereby mental health effects vaccine hesitancy through the mediating variable of vaccine distrust.

**Table 4:** Causal mediation effects.

	effect	std.err	ci.min	ci.max	% share
Total Effect	0.023	0.017	-0.01	0.056	100
Direct Effect	0.008	0.016	-0.024	0.04	35
Indirect Effect	**0.015**	**0.002**	**0.011**	**0.019**	**65**

We are therefore able to conclude that although there is little evidence of current mental distress having a direct effect on vaccine hesitancy, it nevertheless indirectly drives vaccine behaviour through the mediating variables of vaccine distrust. The significant indirect effect accounts for 65% of the total effect. Despite this the total effect is not statistically significant. Nevertheless, this analysis of vaccine distrust as the mediating variable, we have established that indirect effects are highly relevant and need to be considered closely while analysing the relationship between mental distress and vaccine behaviour.

## 5. Findings

This study reveals that vaccine distrust is the most important predictor of vaccine hesitancy. There is little evidence of significant association of either pre-pandemic mental distress or current depressive symptoms with vaccine hesitancy in a fully specified logit model. However, it is clear that mental distress is closely correlated with vaccine distrust.

The mediation regression establishes that depressive symptoms affect vaccine hesitancy via vaccine distrust. The findings indicate that individuals at high risk of depression are more concerned regarding the safety of vaccines, which in turn feeds into vaccine hesitancy. Therefore, depressive symptoms impact on vaccine hesitancy through the mediating factor of vaccine distrust. This finding is robust to the gradient boosted causal mediation model which establishes strong and significant indirect effects, whereby mental health effects vaccine hesitancy through the mediating variable of vaccine distrust.

Despite this, the total effect of mental distress on vaccine hesitancy was not found to be statistically significant. This could be because there are other mediating factors that need to be considered. For eg; depressive symptoms can have a negative effect via the mediating role of risk perception. It can be argued that mental distress enhances risk perception, and this in turn reduces vaccine hesitancy. The opposing effects of different mediating variables like risk perception and vaccine distrust can imply that the net effect of mental distress on vaccine hesitancy is rendered statistically insignificant in the analysis. It is therefore not surprising that the net direct effect of depressive symptoms is insignificant on vaccine hesitancy. Future research therefore should take into account the multiple pathways between mental distress and vaccine hesitancy. Nevertheless, this study has established the role of the mediating variable of vaccine distrust in driving the relationship between mental distress and vaccine behaviour.

## Supporting information

S1 Data (ZIP)
